# REHABILITATION OUTCOMES AS A FUNCTION OF PATIENTS' AND HEALTHCARE PERSONNEL'S VIEWS OF AGING

**DOI:** 10.1093/geroni/igac059.899

**Published:** 2022-12-20

**Authors:** Amit Shrira

**Affiliations:** Bar-Ilan University, Ramat Gan, HaMerkaz, Israel

## Abstract

The effect of subjective views of aging (VoA) on health outcomes is well established, yet this effect was rarely examined among older adults undergoing hospitalization or rehabilitation. Moreover, the additive effect of healthcare personnel’s ageist attitudes on treatment outcomes is unknown. Accordingly, these effects were examined within older adults hospitalized for osteoporotic fractures – a frequent late-life condition with cardinal functional implications. Study 1 (N=147 patients, mean age=79.3) found that feeling younger at admission predicted better rehabilitation outcomes. The reverse effect of functional independence at admission on subjective age at discharge was non-significant. Study 2 (N=52 patients, mean age=81.8, and N=55 hospital staff members, mean age=32.3) replicated these findings with additional VoA indices, and further demonstrated that rehabilitation outcomes were better when occupational and physical therapists reported low levels of ageist attitudes. Findings suggest that successful rehabilitation may be promoted by reducing negative VoA among patients as well as healthcare personnel.

